# Identifying Plant Pentatricopeptide Repeat Coding Gene/Protein Using Mixed Feature Extraction Methods

**DOI:** 10.3389/fpls.2018.01961

**Published:** 2019-01-10

**Authors:** Kaiyang Qu, Leyi Wei, Jiantao Yu, Chunyu Wang

**Affiliations:** ^1^College of Intelligence and Computing, Tianjin University, Tianjin, China; ^2^College of Information Engineering, North-West A&F University, Yangling, China; ^3^School of Computer Science and Technology, Harbin Institute of Technology, Harbin, China; ^4^Department of Electrical Engineering and Computer Science, University of Missouri, Columbia, MO, United States

**Keywords:** pentatricopeptide repeat, mixed feature extraction methods, maximum relevant maximum distance, random forest, J48, naïve bayes

## Abstract

**Motivation:** Pentatricopeptide repeat (PPR) is a triangular pentapeptide repeat domain that plays a vital role in plant growth. In this study, we seek to identify PPR coding genes and proteins using a mixture of feature extraction methods. We use four single feature extraction methods focusing on the sequence, physical, and chemical properties as well as the amino acid composition, and mix the features. The Max-Relevant-Max-Distance (MRMD) technique is applied to reduce the feature dimension. Classification uses the random forest, J48, and naïve Bayes with 10-fold cross-validation.

**Results:** Combining two of the feature extraction methods with the random forest classifier produces the highest area under the curve of 0.9848. Using MRMD to reduce the dimension improves this metric for J48 and naïve Bayes, but has little effect on the random forest results.

**Availability and Implementation:** The webserver is available at: http://server.malab.cn/MixedPPR/index.jsp.

## Introduction

Pentatricopeptide repeat (PPR) proteins include tandem repeats of degenerate 35-amino-acid motifs (PPR motifs) (Chen et al., 2018; Rojas et al., 2018). They form a class of nuclear-encoded proteins arranged in series by multiple repeating units (Li and Jiang, 2018). PPR proteins play a vital role in plant growth and development, and are widely found in eukaryotes and terrestrial plants (Ruida et al., 2013; Wang et al., 2018a). The majority of PPR proteins have mitochondrial or chloroplast localization sequences at the N-terminus, making them an ideal model for studying plant cytoplasmic and nuclear interactions (Wang et al., 2008b). Because of the importance of PPR, this study uses machine learning methods to predict sequences in this class of protein.

As PPRs are proteins, protein prediction methods are applicable to PPR. To predict proteins, some algorithm must be employed to extract features from the sequences. With the development of bioinformatics, many feature extraction methods have been developed. The extraction methods are divided into two categories. Based on amino acid composition, only consider the sequence information and the properties of the amino acids. The second, based on protein structure, considers both sequence information and spatial structure information. The N-gram model is a probabilistic language model based on the Markov assumption (Zhu et al., 2015; Lai et al., 2017; Wei et al., 2017a). Chou et al. (Chou, 2010) proposed a method based on the pseudo amino acid composition (Pse-AAC) that has since been used to predict various protein attributes, such as structural class (Sahu and Panda, 2010; Zhu et al., 2018), subcellular location (Wang et al., 2008b; Yang et al., 2016), essential protein (Sarangi et al., 2013), protein secondary structural content (Chen et al., 2009), T-cell epitope (Zhang et al., 2015), and protein remote homology (Liu et al., 2013, 2015a, 2016a). Liu et al. (2014) enhanced this method by reducing the amino acid alphabet profile, and proposed the physicochemical distance transformation (PDT) (Liu et al., 2012), which is similar to PseAAC. The position-specific scoring matrix (PSSM) (Jones, 1999; Kong et al., 2017) contains abundant evolutionary information and is generated by the Position-Specific Iterated Basic Local Alignment Search Tool (PSI-BLAST) (Altschul and Koonin, 1998; Altschul et al., 1998). Kumar et al. (2007) were able to extract features according to amino acid or dipeptide composition, PSSM, and four-part amino acid compositions. Classifiers such as support vector machines, random forests, and artificial neural networks can be applied to the extracted features.

**Figure 1:**
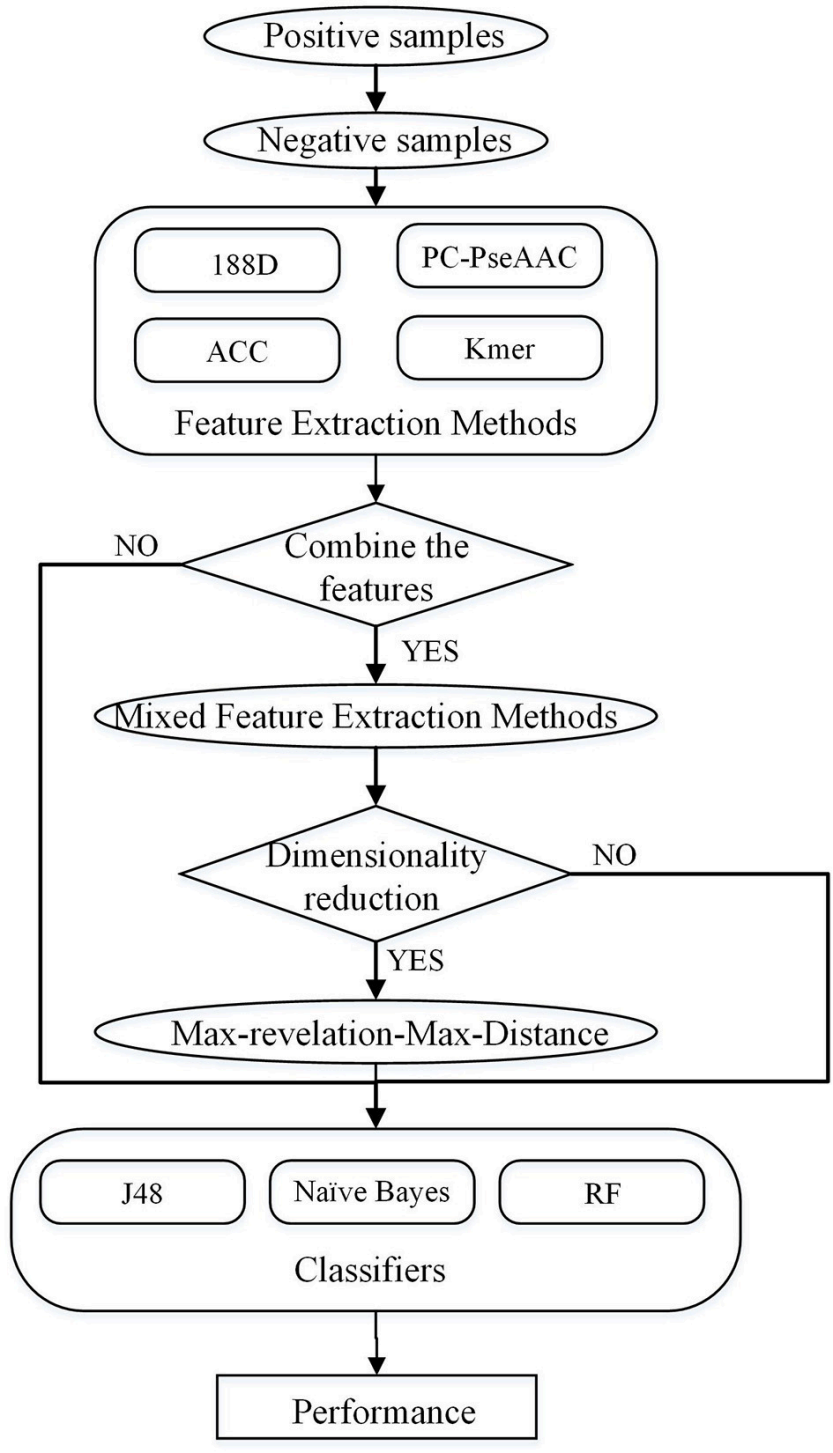
Overall process of the method described in this paper.

In this study, four feature extraction methods and three classifiers are used to predict PPR proteins. The four feature extraction methods not only consider sequence information, but also include the properties of amino acids. We combine these feature extraction methods, and then use the Max-Relevance-Max-Distance (MRMD) method to reduce the dimension. The overall process is shown in Figure 1.

## Methods

### Dataset

For this study, a dataset was extracted from UniPort using the key word “pentatricopeptide repeat” to search the sequences. This search produced 534 reviewed samples, which we used as the positive set. Based on this positive set, we then constructed a negative set as follows. First, we found the Uniport ID of proteins, which have the following symbol: |. Second, we used the Uniport ID to query the proteins' PFAM family. Each sequence belongs to a PFAM family, and similar sequences belong to the same family. After finding all the PFAM families of the PPR positive samples, duplicate PFAM families were deleted to obtain a non-repeating positive family set. We then deleted the positive samples in all families, leaving a set of negative families. Finally, we used the longest protein sequence in each negative family as the negative samples. From the above steps, we obtained 21,960 negative sequences. As some sequences may be redundant, we used CD-HIT (Fu et al., 2012) to reduce the data with a threshold of 0.7 and deleted sequences that included illegal characters. The final dataset contained 487 positive samples and 9,590 negative samples.

To overcome this imbalance in the dataset, we randomly extracted 10 sets of negative samples, and averaged the results of 10 experiments using these 10 sets. Among the negative sequences, the longest had 35,214 amino acids and the shortest had 11 amino acids. The positive sequences ranged from 196 to 1,863 amino acids in length. Thus, we divided the negative samples into four parts according to their length, and extracted 487 sequences from these four parts in proportion.

### Feature Extraction Methods

#### Based on Sequence, Physical, and Chemical Properties

**Figure 2:**
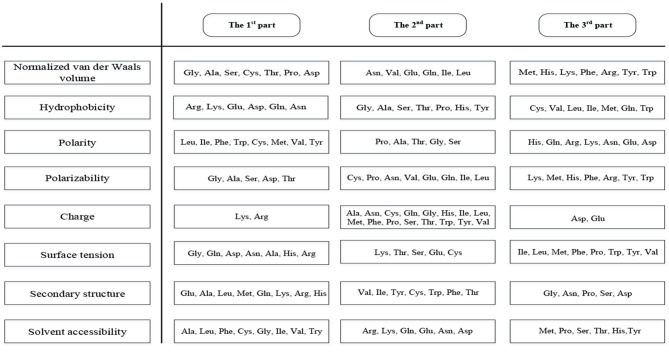
Three groups of amino acids divided according to properties.

This method can extract 188 features (hereinafter referred to as 188D) covering sequence information and amino acid properties (Zhang et al., 2012; Song et al., 2014; Xu et al., 2014). The first 20 features are the frequency of 20 amino acids in the protein sequence. Furthermore, the content, distribution, and dipeptide composition are essential in protein predictions (Song et al., 2014). We divided the 20 amino acids into three groups according to their properties which were shown in Figure 2.

The amino acids were divided into three groups according to their properties, and then we calculated the proportion of the three groups in the sequences for eight properties, giving 3 × 8 = 24 features to be extracted (Cai et al., 2003; Lin et al., 2013). Next, we identified the distribution of the three groups of amino acids at five positions (beginning, 25, 50, 75, and end), giving a further 3 × 5 × 8 = 120 features to be extracted (Cai et al., 2003). Finally, we calculated the number of the three types of dipeptides containing two amino acids from different groups, so another 3 × 8 = 24 features will be extracted. Therefore, the algorithm produces 20 + 24 + 120 + 24 = 188 features (Lin et al., 2013).

#### Pse-in-One

The other three methods are implemented by Pse-in-one, which was proposed by Liu (Liu et al., 2015b) and BioSeq-Analysis (Liu, 2018). We briefly introduce these methods in this section.

##### Kmer

Similar to the N-gram model, kmer extracts features using the amino acid spacer. This method uses the frequency of *k* adjacent amino acid fragments to reflect the sequence composition of the protein. Since there are 20 possibilities for each position, 20^*k*^ features can be extracted. For example, when *k* = 2, the feature is the frequency of amino acid fragments that have two amino acids in the sequence. It can be expressed as follows (Liu et al., 2008):

$$\begin{array}{l} F_{kmer}=\left\{f_{1}^{kmer},f_{2}^{kmer},\cdots f_{20^{k}}^{kmer}\right\} \end{array}$$

##### Auto-cross covariance

The auto-cross covariance (ACC) transforms the protein sequence to a certain length by measuring the relationship between any two properties of the amino acids (Dong et al., 2009). ACC includes two parts: the auto covariance (AC) calculates the relevance of the same property between two residues along sequence intervals of length *lg* (Dong et al., 2009), and the cross-covariance (CC) measures the differences between two properties (Guo et al., 2008). For a protein sequence *P*, the transformation can be written as (Liu et al., 2016b):

$$\begin{array}{l} P^{\prime }={\left[\varphi_{1},\varphi_{2},\cdots \;,\varphi_{N^{*}\mathrm{lg}}\right]}^{T} \end{array}$$

where *N* represents the number of amino acid properties and φ_*n*_ is calculated as (Liu et al., 2016c):

$$\begin{array}{l} \varphi_{n}=AC\left(i,\text{lg}\right)=\frac{1}{N\;-\;\mathrm{lg}}\overset{L-\mathrm{lg}}{\underset{j=1}{\sum }}\left(S_{i,j}-\bar{S_{i}}\right)\left(S_{i,j+lg}-\bar{S_{i}}\right) \end{array}$$

CC transforms the sequence to the vector set:

$$\begin{array}{l} P^{\prime }={\left[\varphi_{1},\varphi_{2},\cdots \;,\varphi_{N^{*}{\left(N-1\right)}^{*}lg}\right]}^{T} \end{array}$$

and then calculates (Guo et al., 2008):

$$\begin{array}{l} \text{CC}\left(\text{i}1,\text{i}2,\text{lg}\right)=\frac{1}{N\;-\;lg}\overset{L-lg}{\underset{j=1}{\sum }}\left(S_{i1,j}-\bar{S_{i1}}\right)\left(S_{i2,j+lg}-\bar{S_{i2}}\right) \end{array}$$

where *i* denotes the residues, *L* represents the length of the sequence, *S*_*i, j*_ is the score of the *j*-th amino acid with respect to the *i*-th property, and $\bar{S_{i}}$ is the average score for *i* along the sequence.

In this study, we selected three properties and set *lg* = 2.

##### Parallel correlation pseudo amino acid composition

Parallel correlation pseudo amino acid composition (PC-Pse-AAC) considers composition, properties, and sequence orders (Chou, 2010; Xiao and Chou, 2011).

We consider a protein sequence *P* containing *L* amino acids. The sequence can be represented by 20 + λ features as:

$$\begin{array}{l} FV_{PseACC}={\left[x_{1},x_{2},\ldots ,x_{20+\lambda }\right]}^{T} \end{array}$$

where λ is a distance parameter that reflects the effect of the amino acid sequence-order (Pan G. et al., 2018).

The first 20 features are the frequencies at which 20 amino acids appear in the sequence. The other features are given by (Mei and Zhao, 2018):

$$\theta_{k}\;=\frac{\sum_{i=1}^{L-k}\Theta (A_{i},A_{i+k})}{L-k}\;(\text{k}\leq \lambda )$$

$$\Theta \left(A_{i},A_{i+k}\right)=\frac{1}{T}\sum_{j=1}^{T}{\left(I_{j}\left(A_{i}\right)-I_{j}\left(A_{i+k}\right)\right)}^{2}$$

$$I_{j}\left(A_{i}\right)\;=\frac{I_{j}^{\prime }\left(A_{i}\right)-\sum_{m=1}^{20}\frac{I_{j}^{\prime }\left(R_{m}\right)}{20}}{\sqrt{\frac{{\sum_{k=1}^{20}\left(I_{j}^{\prime }\left(R_{k}\right)-\sum_{m=1}^{20}\frac{I_{j}^{\prime }\left(R_{m}\right)}{20}\right)}^{2}}{20}}}$$

where *A*_*i*_ represents the *i*-th amino acid in the protein sequence, and *k* denotes the distance between two amino acids along the protein sequences. *T* is the number of physicochemical properties, and *I*_*j*_(*A*_*i*_) is the *j*-th property of $A_{i}.\;I_{j}^{\prime }\left(A_{i}\right)$ indicates the original physicochemical property score of amino acid *A*_*i*_ with respect to property *j*, and *R*_*m*_ represents the 20 amino acids.

In this study, we selected three properties and set λ = 2.

#### Mixed Feature Extraction Methods

The Max-Relevance-Max-Distance (MRMD) (Zou et al., 2016; Qu et al., 2017; Wei et al., 2017b) technique was used to reduce the dimension. We used the Pearson correlation coefficient (PCC) to measure the relevance and the Euclidean distance function to identify instances of redundancy.

The PCC can calculate continuous variables and is easy to implement. Therefore, the PCC (Ahlgren et al., 2014) was used to measure the relationship between the features and the target class in the MRMD feature dimension reduction method. The formula for the PCC is (Zou et al., 2016):

$$\begin{array}{l} \text{PCC}\left(\vec{X},\vec{Y}\right)=\frac{\frac{1}{N-1}\sum_{k=1}^{N}\left(x_{k}-\bar{x}\right)\left(y_{k}-\bar{y}\right)}{\sqrt{\frac{1}{N-1}\sum_{k=1}^{N}{\left(y_{k}-\bar{y}\right)}^{2}}\sqrt{\frac{1}{N-1}\sum_{k=1}^{N}{\left(x_{k}-\bar{x}\right)}^{2}}} \end{array}$$

where *x*_*k*_ represents the *k*th element in $\vec{X}$, and $\vec{X}$, $\vec{Y}$ are vectors composed of each instance's features. Thus, the maximum relevance of the *i*th feature is:

$$\begin{array}{l} \max MR_{i}=|\text{PCC}\left(\vec{F_{i}},\vec{C_{i}}\right)| \end{array}$$

The Euclidean distance is given by:

$$\begin{array}{lll} \text{ED}\left(\vec{X},\vec{Y}\right) & = & \sqrt{\overset{N}{\underset{k=1}{\sum }}{\left(x_{k}-y_{k}\right)}^{2}}\\ \max MD_{i} & = & \text{E}D_{i}=\frac{1}{M-1}\sum \text{ED}\left(\vec{{\text{F}}_{\text{i}}},\vec{F_{k}}\right) \end{array}$$

We selected features according to:

$$\begin{array}{l} \max\left(MR_{i}+MD_{i}\right) \end{array}$$

As the PCC increases, the relationship between the features and the target classes becomes stronger. The greater the distance between features, the less redundancy exists in the vectors. The final feature set created by this method has less redundancy and greater correlation with the target set (Xu et al., 2016, 2018; Jiang et al., 2017; Wei et al., 2017c).

## Feature selection method

### Classifiers

We used three classifiers in this study: random forest (RF), naïve Bayes (NB), and J48. The classifiers can be implemented in WEKA, which is based on the Java environment.

#### J48

The J48 method is a decision tree algorithm based on C4.5 (Mohasseb et al., 2018). Decision trees (Quinlan, 1986) are a graphical approach using probability analysis. J48 is a kind of supervised learning, whereby each sample has a set of attributes and a predetermined label. By learning about the samples, a classifier can be taught to generate classification results for new instances (Rondovic et al., 2019).

In each step, decision trees select an attribute to split. Ideally, the optimal attribute should be selected so that the samples included in the branch nodes of the decision tree belong to the same class (Kothandan and Biswas, 2016; Zhong et al., 2018). The selection of attributes is an important problem, and many methods have been derived for this purpose, such as information gain, and information gain ratio. The C4.5 method uses the information gain ratio to select which attributes to split.

#### Random Forest

Ensemble learning is an effective technique that has been applied to many fields of bioinformatics (Li et al., 2016; Liu et al., 2016d, 2018; Zhang et al., 2016a; Tang et al., 2017; Pan Y. et al., 2018; Wang H. et al., 2018; Wei et al., 2018a,b). The RF approach (Wang S. P. et al., 2018) is an ensemble learning method that employs many decision trees, with the output result dependent on “votes” cast by each tree. The construction process is as follows.

First, we determine the quantity of decision trees (*m*), the depth of each tree (*d*), and the number of features (*f*) used by each node. Then, *n* samples are selected at random from the samples set. In addition, *f* features are randomly selected, and the selected samples use these features to build decision trees. This step is repeated *m* times to give *m* decision trees, forming the random forest. Each decision tree classifies each sample, so each decision tree outputs a value. For classification problems, the final result is the class that has the most votes. For regression problems, the final result is the average of the output of all decision trees (Song et al., 2017).

#### Naïve Bayes

NB (Rajaraman and Chokkalingam, 2014; Deng and Chen, 2015) is a classical classifier based on conditional probability. The most important component of NB is the Bayesian rule, which is given by (Yu et al., 2015):

$$\begin{array}{l} \text{p}\left(B_{i}|A\right)=\frac{p\left(A|B_{i}\right)\;p\left(B_{i}\right)}{\sum_{j=1}^{n}p\left(A|B_{j}\right)\;p\left(B_{j}\right)} \end{array}$$

where p(*B*_*i*_|*A*) represents the conditional probability of event *B*_*i*_ occurring under event *A*. *p*(*B*_*i*_) is the marginal probability of independent event *B*_*i*_.

The classification principle is that use the Bayesian rule to calculate the posterior probability of an object based on its prior probability, and then select the class with the largest posterior probability as the class to which the object belongs. In this method, all features are statistically independent. So according to the above formula, we can get the following formula:

$$\begin{array}{l} \text{p}\left(y|x_{1},\cdots \;,x_{n}\right)=\frac{p\left(y\right)\prod_{i=1}^{n}p\left(x_{i}|y\right)}{p\left(x_{1}\right)p\left(x_{2}\right)\cdots p\left(x_{n}\right)} \end{array}$$

Then, the above formula can be converted into:

$$\begin{array}{l} ŷ=arg\underset{y}{\max}p\left(y\right)\overset{n}{\underset{i=1}{\prod }}p\left(x_{i}|y\right) \end{array}$$

Where, *y* represents class variables and *x*_*i*_ represents features. ŷ represents the predicted class.

### Measurement

As we have an imbalanced dataset, we use the area under the receiver operating characteristic (ROC) curve (AUC) and the F-Measure to evaluate the performance of the classifiers.

The abscissa of the ROC curve is the false positive rate (FPR), and the ordinate is the true positive rate (TPR). AUC is the area under the ROC curve, which always has a value of less than one (Lobo et al., 2010; Pan et al., 2017; Wei et al., 2018d). As the ROC curve is generally above the straight line *y* = *x*, the value of AUC tends to be greater than 0.5 (Fawcett, 2005). The larger the value of AUC, the better the classification performance.

The F-measure (Nan et al., 2012) is a weighted harmonic average of precision and recall. This metric, which is often used to evaluate the quality of classification models, is computed as follows:

$$\begin{array}{lll} \text{precision} & = & \frac{TP}{TP\;+\;FP}\\ \text{recall} & = & \frac{TP}{TP\;+\;FN}\\ \text{F}-\text{measure} & = & \frac{\left(\alpha^{2}\;+\;1\right)precision^{*}recall}{\alpha^{2}\left(precision\;+\;recall\right)} \end{array}$$

Typically, α = 1, so that:

$$\begin{array}{l} \text{F}1=\frac{2precision^{*}recall}{precision\;+\;recall} \end{array}$$

## Results and Discussion

Experiments were conducted using 10-fold cross-validation (Wei et al., 2018c; Zhao et al., 2018), whereby the dataset is divided into 10 sections, with nine parts used to train the model and the remaining one used for testing. This process is repeated 10 times, and the average of all the tests gives the final result.

### Results Using Individual Feature Extraction Methods

In this section, we discuss the performance of each individual feature extraction method. The four feature extraction methods focus on different aspects. 188D considers information about the sequence composition and amino acid properties, whereas kmer considers the frequency of amino acid fragments in the sequence. ACC considers three properties, hydrophobicity, hydrophilicity, and mass, and PC-PseAAC considers the amino acids' distance and properties. Table 1 presents the results using these methods with each classifier.

**Table 1:** PPR prediction results using a single feature extraction method.

**Method**	**Classifier**	**AUC**	**F-Measure**
188D	RF	**0.9788**	**0.9448**
	J48	0.8684	0.8786
	Naïve bayes	0.907	0.8192
Kmer	RF	**0.9826**	**0.9492**
	J48	0.8284	0.8312
	Naïve bayes	0.9162	0.8344
Acc	RF	**0.9524**	**0.8898**
	J48	0.8456	0.8406
	Naïve bayes	0.9428	0.8594
PC-PseAAC	RF	**0.9752**	**0.9366**
	J48	0.8710	0.8740
	Naïve bayes	0.9678	0.9076

**Figure 3:**
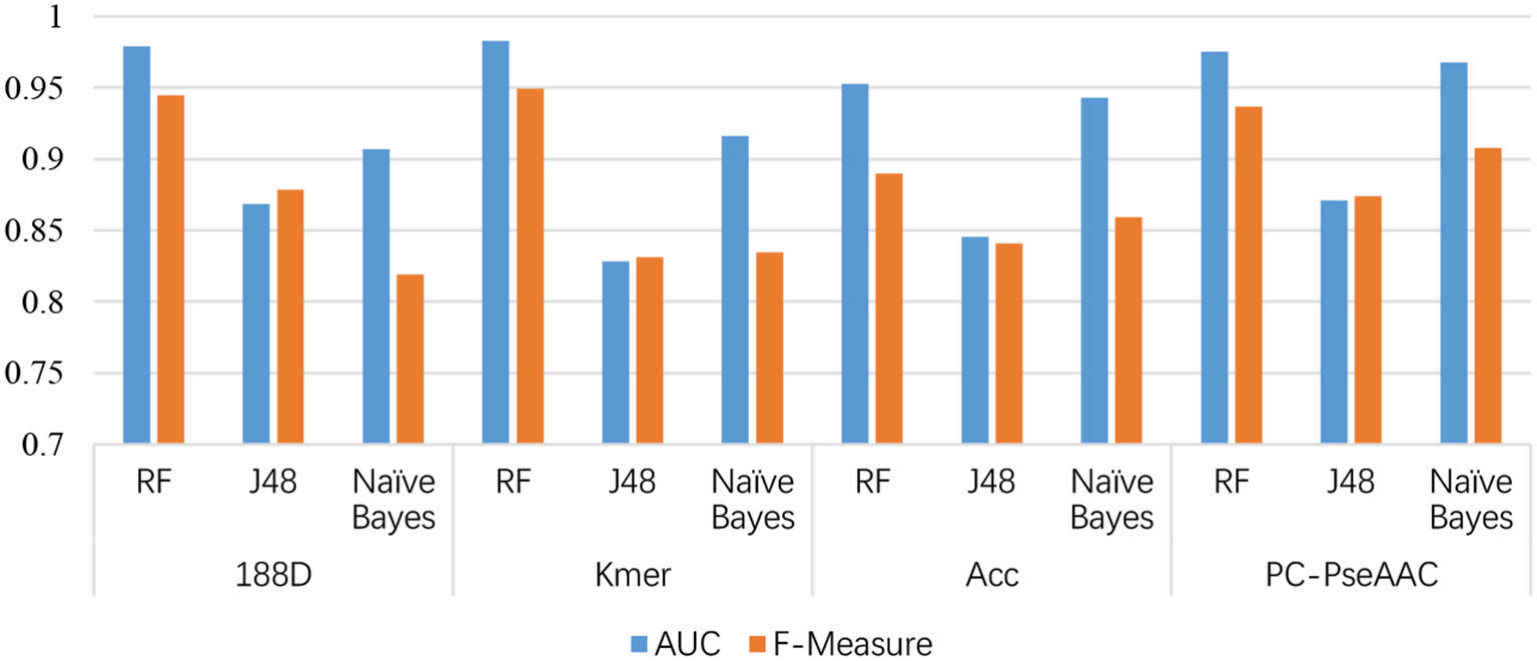
Comparison of AUCs among the four feature extraction methods and three classifiers.

*To represent the experimental results more intuitively, they are displayed as a histogram in Figure 3. Bold values indicates Best result in that experiment results which is a combination of Method and Classifier*.

From Table 1, it is clear that the performance is generally good. RF produced the best performance, especially with the kmer feature extraction method, achieving an AUC score of 0.9826. J48 has the worst performance, although this method attained an AUC score of 0.8710 when used with PC-PseAAC. NB performed best with the PC-PseAAC feature extraction method. Obviously, RF is better than J48. This may be because the random forest uses results from multiple decision trees, thus avoiding some exceptional cases.

### Performance of Joint Feature Extraction Methods

Next, we connected the feature extraction methods to give six new feature sets: 188D + ACC (206D), 188D + kmer (588D), 188D + Pse-AAC (210D), ACC + kmer (418D), ACC + Pse-AAC (40D), Pse-AAC + kmer (422D).

Table 2 presents the results given by mixing the features. And we add the best performance of single into Table 2, which can make a more intuitive comparison. From the table, we can see that the performance using the RF classifier is slightly better than for the single 188D method. The highest AUC is 0.9820 and the lowest AUC is 0.8554.

**Table 2:** Results from mixing the features.

**Method**	**Classifier**	**AUC**	**F-measure**
Kmer	RF	0.9826	0.9492
188D + ACC	RF	**0.9820**	**0.9520**
	J48	0.8868	0.8886
	Naïve bayes	0.9150	0.8294
188D + kmer	RF	**0.9814**	**0.9494**
	J48	0.8554	0.8608
	Naïve bayes	0.9088	0.8340
188D-Pse-AAC	RF	**0.9796**	**0.9490**
	J48	0.8806	0.8866
	Naïve bayes	0.9174	0.8368
ACC + kmer	RF	**0.9848**	**0.9554**
	J48	0.8518	0.8538
	Naïve bayes	0.9252	0.8516
PseAAC + kmer	RF	**0.9826**	**0.9504**
	J48	0.8386	0.8446
	Naïve bayes	0.9252	0.8532
ACC + Pse-AAC	RF	**0.9778**	**0.9402**
	J48	0.8632	0.8748
	Naïve bayes	0.9736	0.9214

*Bold values indicates Best result in that experiment results which is a combination of Method and Classifier*.

Next, we combined kmer with another method. The results are presented in Table 2. In this case, the best AUC is 0.9848 and the lowest AUC is 0.8386, which are both higher than the scores achieved using the kmer method alone. RF gives the best performance, and J48 is again the worst classifier.

The results from combining Pse-AAC with another method are presented in Table 2. We can see that the overall performance is worse than in the above cases. With the exception of the RF results, the performance is worse than when using the Pse-AAC method on its own. In this case, the best AUC score is 0.9826 and the worst is 0.8386.

The results from combining ACC with another method are shown in Table 2. Compared with the results using ACC alone, the performance has improved, except when using the NB classifier. RF again gives the best results and J48 gives the worst. The highest AUC score is 0.9848 and the lowest is 0.8518.

From the above results, we can conclude that RF is the best classifier for this task, whereas J48 is unsuitable in this case. The best PPR prediction method is to combine ACC and kmer and use the RF classifier, which achieves the highest AUC of 0.9848.

### Performance Using MRMD to Reduce the Dimension

Next, we used MRMD to reduce the dimension of the features considered in section Performance of Joint Feature Extraction Methods, resulting in six new feature sets. As the features were randomly extracted from the dataset 10 times, the number of features after dimension reduction was inconsistent. We conducted experiments using 10 separate sets of data. We then selected the feature set with the best AUC performance and applied this feature set to the remaining nine datasets. The final results are the average of 10 experiments.

**Figure 4:**
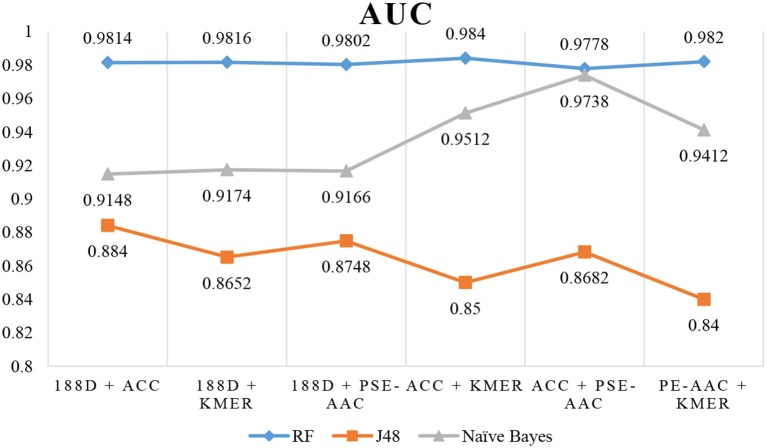
AUC when using MRMD to reduce the dimension.

**Figure 5:**
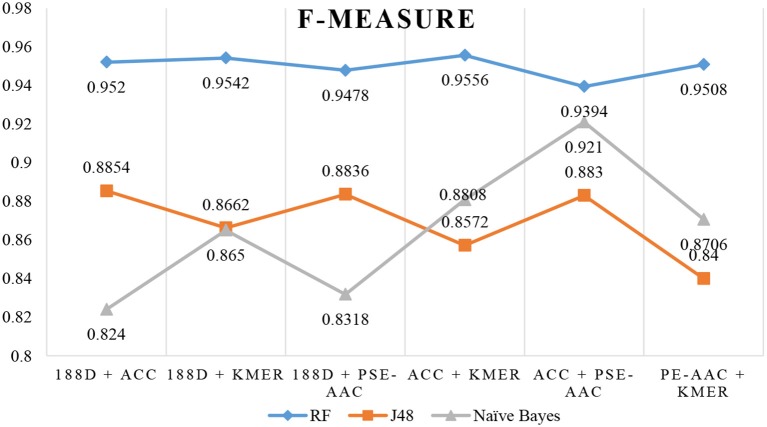
F-Measure when using MRMD to reduce the dimension.

The results are shown in Table 3, Figures 4, 5. The highest AUC value is 0.9840, and the lowest is 0.8400. Again, RF gives the best performance and J48 is the worst classifier. From the figures, although J48 has the worst performance, the AUCs have improved. In particular, using MRMD for dimension reduction results in better performance by the NB classifier.

**Table 3:** Results from reduction the features.

**Method**	**Classifier**	**AUC**	**F-Measure**
188D + ACC	RF	**0.9814**	**0.9520**
	J48	0.8840	0.8854
	Naïve bayes	0.9148	0.8240
188D + kmer	RF	**0.9816**	**0.9542**
	J48	0.8652	0.8662
	Naïve bayes	0.9174	0.8650
188D-Pse-AAC	RF	**0.9802**	**0.9478**
	J48	0.8748	0.8836
	Naïve bayes	0.9166	0.8318
ACC + kmer	RF	**0.9840**	**0.9556**
	J48	0.8500	0.8572
	Naïve bayes s	0.9512	0.8808
PseAAC + kmer	RF	**0.9820**	**0.9508**
	J48	0.8400	0.8400
	Naïve bayes	0.9412	0.8706
ACC + Pse-AAC	RF	**0.9778**	**0.9394**
	J48	0.8682	0.8830
	Naïve bayes	0.9738	0.9210

*Bold values indicates Best result in that experiment results which is a combination of Method and Classifier*.

## Conclusion

PPR proteins play an important role in plants. In this study, we used machine-learning methods to predict this type of protein. To find the best performance, we used four feature extraction methods that consider sequence, physical, and chemical properties as well as the amino acid composition, and three classifiers. In terms of the individual feature extraction methods, using kmer with the RF classifier gave the highest AUC. Next, we combined the feature extraction methods, and found that RF still achieved the best performance while J48 gave the worst results. Finally, we used MRMD to reduce the feature dimension. This improved the AUCs for the J48 and NB classifiers, but had little effect on the RF results. The highest AUC score of 0.9848 was achieved by combining ACC and kmer and using RF as the classifier. The webserver is freely available at: http://server.malab.cn/MixedPPR/index.jsp. In future work, it can be expected to further improve the performance by integrating other informative features such as motif-based features (Li et al., 2010; Ma et al., 2013; Yang et al., 2017), and validate the reliability of our method using next-generation sequencing analysis (Zhang et al., 2016b; Liu et al., 2017).

## Author Contributions

KQ implemented the experiments and drafted the manuscript. LW and CW initiated the idea, conceived the whole process, and finalized the paper. KQ and JY helped with data analysis and revised the manuscript. All authors have read and approved the final manuscript.

### Conflict of Interest Statement

The authors declare that the research was conducted in the absence of any commercial or financial relationships that could be construed as a potential conflict of interest.
